# The Role of Progranulin (PGRN) in the Pathogenesis of Glioblastoma Multiforme

**DOI:** 10.3390/cells13020124

**Published:** 2024-01-10

**Authors:** Łukasz A. Poniatowski, Michał Woźnica, Piotr Wojdasiewicz, Aneta Mela-Kalicka, Katarzyna Romanowska-Próchnicka, Daryush Purrahman, Grzegorz Żurek, Maciej Krawczyk, Najmeh Nameh Goshay Fard, Marzena Furtak-Niczyporuk, Janusz Jaroszyński, Mohammad-Reza Mahmoudian-Sani, Ilona Joniec-Maciejak

**Affiliations:** 1 Department of Neurosurgery, Dietrich-Bonhoeffer-Klinikum, Salvador-Allende-Straße 30, 17036 Neubrandenburg, Germany; 2 Department of Spine Surgery, 7th Navy Hospital, Polanki 117, 80-305 Gdańsk, Poland; michalwoznica.med@gmail.com; 3 Department of Biophysics, Physiology and Pathophysiology, Faculty of Health Sciences, Medical University of Warsaw, Chałubińskiego 5, 02-004 Warsaw, Polandkatarzyna.prochnicka@gmail.com (K.R.-P.); 4 Department of Experimental and Clinical Pharmacology, Centre for Preclinical Research and Technology (CePT), Medical University of Warsaw, Banacha 1B, 02-097 Warsaw, Poland; 5 Department of Systemic Connective Tissue Diseases, Eleonora Reicher National Institute of Geriatrics, Rheumatology and Rehabilitation, Spartańska 1, 02-637 Warsaw, Poland; 6 Thalassemia and Hemoglobinopathy Research Center, Health Research Institute, Ahvaz Jundishapur University of Medical Sciences, Ahvaz, Iran; daryushpurrahman@gmail.com (D.P.);; 7 Department of Biostructure, Wrocław University of Health and Sport Sciences, I. J. Paderewskiego 35, 51-612 Wrocław, Poland; grzegorz.zurek@awf.wroc.pl; 8 2nd Department of Neurology, Institute of Psychiatry and Neurology, Sobieskiego 9, 02-957 Warsaw, Poland; 9 Department of Public Health, Faculty of Medicine, Medical University of Lublin, Chodźki 1, 20-093 Lublin, Poland; 10 Department of Administrative Proceedings, Faculty of Law and Administration, Maria Curie-Skłodowska University of Lublin, Marii Curie-Skłodowskiej 5, 20-031 Lublin, Poland

**Keywords:** progranulin, glioblastoma multiforme, brain tumour, oncogenesis, drug resistance, temozolomide

## Abstract

Glioblastoma multiforme (GBM) represents the most common and aggressive malignant form of brain tumour in adults and is characterized by an extremely poor prognosis with dismal survival rates. Currently, expanding concepts concerning the pathophysiology of GBM are inextricably linked with neuroinflammatory phenomena. On account of this fact, the identification of novel pathomechanisms targeting neuroinflammation seems to be crucial in terms of yielding successful individual therapeutic strategies. In recent years, the pleiotropic growth factor progranulin (PGRN) has attracted significant attention in the neuroscience and oncological community regarding its neuroimmunomodulatory and oncogenic functions. This review of the literature summarizes and updates contemporary knowledge about PGRN, its associated receptors and signalling pathway involvement in GBM pathogenesis, indicating possible cellular and molecular mechanisms with potential diagnostic, prognostic and therapeutic targets in order to yield successful individual therapeutic strategies. After a review of the literature, we found that there are possible PGRN-targeted therapeutic approaches for implementation in GBM treatment algorithms both in preclinical and future clinical studies. Furthermore, PGRN-targeted therapies exerted their highest efficacy in combination with other established chemotherapeutic agents, such as temozolomide. The results of the analysis suggested that the possible implementation of routine determinations of PGRN and its associated receptors in tumour tissue and biofluids could serve as a diagnostic and prognostic biomarker of GBM. Furthermore, promising preclinical applications of PGRN-related findings should be investigated in clinical studies in order to create new diagnostic and therapeutic algorithms for GBM treatment.

## 1. Introduction

Glioblastoma multiforme (GBM) constitutes the most common and aggressive malignant form of primary brain tumour in adults that carries the poorest prognosis and mortality rate [1]. The median overall survival after diagnosis of grade IV astrocytoma, according to the World Health Organization (WHO) classification, amounts to ~15 months. The median patient age at diagnosis is ~64 years, where men are significantly more often diagnosed than women, and only ~5.5% of patients survived 5 years after diagnosis [2]. If occurrence is to be considered from a geographical point of view, in the United States of America (USA), the incidence rate of GBM is 3.19 per 100,000 people, with a median age of 64 years. It seems that the precise gender-dependent incidence is 1.6 times higher in males compared to females [3]. According to these data, a higher percentage of the Caucasian population tends to suffer from more GBM cases compared to Africans and Afro-Americans, with a lower incidence in Asians and American Indians. This may be the reason why European Union (EU) countries like Germany (~5.77 cases per 100,000), France (~3.3 cases per 100,000) and Great Britain (~3.27 cases per 100,000) tend to have more diagnosed cases per year than Asia or Latin America and the Caribbean [4]. Another view that is supported by the American Association of Neurological Surgeons (AANS) is based on the observation that developed countries tend to have sufficient medical infrastructure to diagnose more cases, hence the greater occurrence in the USA and EU [5]. Therapy places a significant cost burden on patients regarding health-related quality of life (HRQoL) and the health system [6]. In recent decades, neuroscientists and oncologists have explored various advances in GBM therapy. Currently, the management of GBM is based on combined maximal safe surgical resection, radiotherapy and chemotherapy with temozolomide, eventually with the addition of adjuvant anti-vascularising agents [7]. Actual concepts of the pathophysiology of cancer pathology, contributing to carcinogenesis and tumour progression are inextricably linked with ongoing local and systemic inflammatory responses [8]. In this case, brain tumours and brain metastases also initiate progressive changes and remodeling of the brain tissue microenvironment, leading to the development of a neuroinflammatory response [9]. The main neuropathological feature of GBM is tissue necrosis, which is connected with the inflammatory component around the tumour region [10]. In this situation, the presence of immunosuppressive inflammation is associated with necrosis and constitutes a feature that is connected with higher resistance to therapy and worse prognosis [11]. GBM constitutes cells that are characterized by aggressive growth and intense vascularity, corresponding with the synthesis of large amounts of mediators, such as cytokines, chemokines, and growth factors, which promote infiltration and the induction of protumour cellular phenotypes [12]. The ongoing immunosuppressive and neuroinflammatory changes enabled by the synthesis of a number of mediators are consequently responsible for proliferation, migration, angiogenesis and resistance to therapy [13]. Currently, pleiotropic and multifunctional growth factor progranulin (PGRN) has attracted significant attention in the neuroscience and oncological community because of its potent and specific anti-inflammatory, immunomodulatory and neurotrophic features [14]. Two studies published in 2006 suggest that haploinsufficiency and null mutations in the PGRN gene are responsible for the development of several familiar forms of frontotemporal lobar degeneration (FTLD) and sparked landmark studies focused on evaluating the precise role and function of PGRN in brain physiology and pathophysiology [15,16]. Therefore, while PGRN deficiency seems to be associated with a broad range of neurodegenerative brain diseases, its upregulation and high expression are often associated with processes such as tissue repair, embryonic development and tumourigenesis [17]. In this case, the role of PGRN in tumour pathogenesis covers a wide range of molecular activities regarding enhanced cell proliferation, migration, invasion, adhesion, and angiogenesis, as well as the maintenance of cancer stem cells and the tumour microenvironment [18]. Based on the general PGRN expression profile, it should be mentioned that its mRNA transcripts are constitutively present in less differentiated and rapidly proliferative cells in contrast to completely differentiated and mitotically quiescent cells, demonstrating a low proliferation rate in somatic tissues [19,20]. Those findings summarize the role of PGRN as a distinct regulator of cell cycle kinetics and proliferative homeostasis influencing both the S and M phases independently from other growth factors, indirectly explaining its increased expression in a variety of human cancers and tumour cell lines [21,22]. The overexpression of PGRN was observed in several cancer types and cell lines, including ovarian, breast, bladder, liver, adrenal, prostate, kidney, multiple myeloma, leiomyosarcoma and mesothelioma [17]. In addition, the observed overexpression of PGRN could be associated with resistance to various chemotherapeutic agents, such as tamoxifen and cisplatin [23,24,25]. Consequently, the accompanying overexpression of PGRN in tumourigenesis could potentially have diagnostic and prognostic significance. To date, the relatively high mRNA expression of PGRN has been reported in multiple types of gliomas [26]. On the one hand, it seems that PGRN serves as a physiological regulator of neuronal and neurothropic functions that regulates neurite outgrowth and maintains its survival, and on the other hand, growing knowledge about PGRN and its associated multiple pathways suggest that, in cancer, PGRN constitutes a distinct, critical molecule associated with pathogenesis. Despite this dual role of PGRN, its position has not been widely discussed in the context of its presence and possible functions in the pathophysiology of GBM-related phenomena. It seems justified to describe and summarize the participation of PGRN in the course of GBM from the available relevant literature. In order to present a comprehensive overview of the problem, we performed a thorough analysis of the structure and functions of PGRN, presented its role in the physiological processes related to tumourigenesis in the context of brain tissue and discussed the possible role in the course of GBM with particular attention paid to each stage of the pathology and consequences and also possible new therapeutic modalities.

## 2. Biological Functioning and Molecular Aspects of PGRN

### 2.1. PGRN as Unique Multifunctional Growth Factor

**Figure 1:**
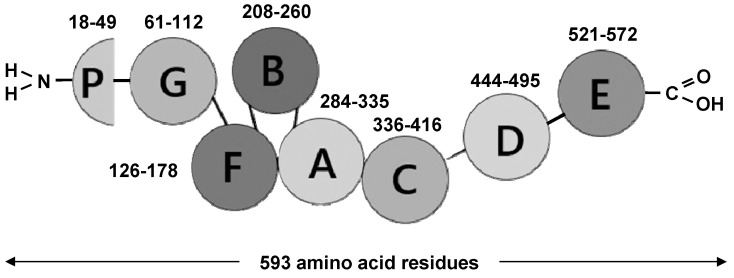
The schematic representation of progranulin (PGRN) structure. Based on the ultrastructural studies, the intact molecule protein includes seven (A–G) and a half (P) tandem repeats of a cysteine (Cys)-rich motif situated as individual granulins (GRN) in a sequential order, which are presented as letters in polypeptide chain.

The multistep discovery of PGRN was the result of investigations performed by several independent scientific groups in various context according to the chosen methodology of the performed experiment. In 1990, Shoyab et al. first identified and isolated two single-chain cysteine (Cys)-rich (~20%) proteins (~6 kDa) from rat kidneys and named them epithelin (EPI) 1 (EPI-1) and epithelin 2 (EPI-2) [27]. In the same year (at the interval of 2–3 months), Bateman et al. confirmed and described the existence of the novel protein family, which was obtained from human peripheral leukocyte granule extracts, calling them granulin (GRN) A (GRN-A), -B (GRN-B), -C (GRN-C) and -D (GRN-D) [28]. The fifth related protein isolated and partially sequenced from rat bone marrow (rat granulin) was identical to human GRN-A. The group of proteins discovered also shared similar properties due to the highly conserved molecular structure and Cys-like activities. In this case, EPI-1 and EPI-2 shared identity with human GRN-A and GRN-B [27,28]. In 1992, both scientific groups (at the interval of 3–4 months) performed complementary DNA (cDNA) analysis and cloned the gene responsible for the translation of a single large precursor protein (prepropeptide) containing 7.5 EPI/GRN motifs [29,30]. Further investigations, including large-scale biochemical fractionation carried out by other laboratories in the following years, led to the isolation of this precursor protein, which was named in relation to a broad range of different biological tissue sources, e.g., PEPI, acrogranin, PCDGF, TGFe, GEP or GP88 [31,32,33,34,35,36,37]. The alternative names reflect some of the functional features of PGRN [21]. In the human genome, the PGRN gene is composed of 12 coding exons (E2–13) and a non-coding exon (E1), covering about 3.700 base pairs (bp) with 10 kb in the protein-coding region and located at 1.7 Mb centromeric of microtubule-associated protein tau (MAPT) within the long arm of chromosome 17 (17q21.31) [15,16]. According to evolutionary dynamics, PGRN seems to be a phylogenetically ancient molecule that evolved only once, about ~1.5 billion years ago, and traced to unicellular organisms, which is in accordance with the distribution and structure of GRN tandem repeats [21,38]. In this case, GRN tandem repeats are found in unicellular eukaryotes, plants, metazoan animals, and many vertebrate lineages and other extant animalia [21,38]. PGRN is translated as a polypeptide chain composed of 593 amino acid (aa) residues (1–593 aa) with a molecular weight (Mr) of approximately ~68.5 kDa, which becomes an 88 kDa form after heavy glycosylation (mainly fucosylated oligosaccharides) [22,33]. The intact form of PGRN includes a polypeptide chain (holoprotein) that encompasses the N-terminal signal peptide (SP) included in 17 aa residues and 7.5 sequentially arranged multiple tandem repeats of a highly conserved 12 Cys-rich motif (CX_5–6_CX_5_CCX_8_CCX_6_CCXDX_2_HCCPX_4_CX_5–6_C), separated by short intervening spacer/linker (P1/2/3/4/5/6/7) sequences in the order p-G-F-B-A-C-D-E (p-P1-G-P2-F-P3-B-P4-A-P5-C-P6-D-P7-E), where A-G represents full repeats and p is a half motif (paragranulin) (Figure 1) [39]. According to the topological superstructure revealed via nuclear magnetic resonance (NMR) spectroscopy, each 12 Cys-rich motif forms six disulphide (S-S) bridges, adopting a 3D stereochemical compact structure of a parallel stack of beta (β)-hairpins stabilized through 44 disulphide bridges in the configuration of a left-handed super-helix [40]. PGRN undergoes intra- and extracellular proteolytic cleavage, leading to the release of several individually liberated GRNs, 56–57 aa residues with a molecular weight of approximately ~6 kDa, which, after proteolysis, occurs both in individual or linked combination forms (~6–25 kDa) [28,30]. The proteolytic cleavage process is mediated by various intra- and extracellular serine (Ser) and threonine (Thr) proteinases, including matrix metalloproteinase 9 (MMP-9), matrix metalloproteinase 12 (MMP-12), matrix metalloproteinase 14 (MMP-14), a disintegrin and metalloproteinase with thrombospondin motifs 7 (ADAMTS-7), neutrophil elastase (ELANE) and proteinase 3 (PRTN3) where the interactions occur in both directions on the basis of a feed-forward loop [41,42,43,44,45]. It is important to underline that the precise regulation of PGRN proteolysis is restricted by binding proteins that include secretory leukocyte protease inhibitor (SLPI), prosaposin (PSAP) and high-density lipoprotein (HDL) apolipoprotein A1 (ApoA1), which bind to PGRN and inhibit its cleavage [46,47].

### 2.2. Overview of PGRN Associated Receptors and Signalling Pathways

**Figure 2:**
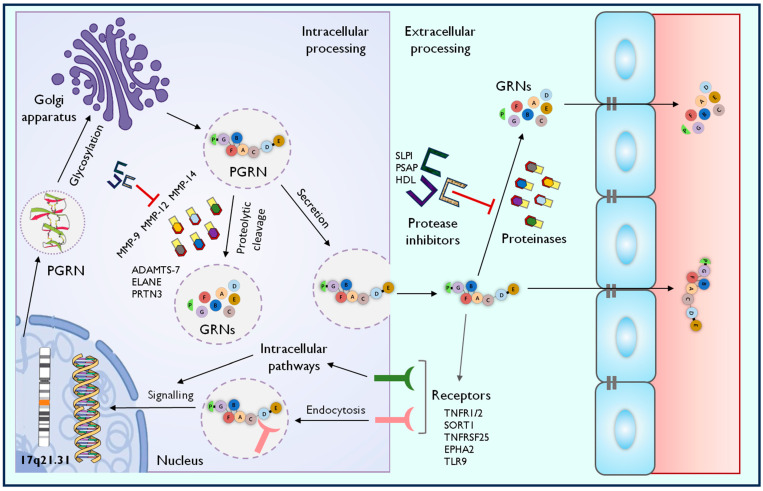
The schematic representation of biological activities and signalling pathways associated with progranulin (PGRN). This representational diagram depicts the biomolecular interactions of PGRN inside and outside the cell.

The identification of the first cell surface receptor for PGRN took more than 20 years after the discovery of EPI-1 and EPI-2, representing a crucial step in understanding the biological activities and signalling pathways associated with PGRN ligand–receptor interactions networks and activities (Figure 2) [27]. The first discovered receptor covers sortilin 1 (SORT1), which binds and interacts with the GRN-E part of PGRN. SORT1 is a multiligand single-pass type I transmembrane protein of the vacuolar protein-sorting 10 (VPS10) family, localized both in the cell surface and endolysosomal compartments [48]. The various biological processes of the SORT1 mainly cover the trafficking of different proteins from the cell surface to intracellular compartments, such as lysosomes and endosomes, through the trans-Golgi network (TGN) in neuronal and non-neuronal cells. In this case, after binding PGRN to SORT1, the whole ligand–receptor complex undergoes endocytosis from the extracellular space, which is associated with the further delivery of PGRN to the lysosomes. The regulation within this signalling axis could be rather regarded as an endogenous regulatory mechanism of extracellular PGRN levels and endocytosis/exocytosis turnover. SORT1, as a multiligand receptor, also participates in the very low-density lipoprotein (VLDL) secretion and proneurotrophin (proNT)-induced apoptosis; likewise, it is responsible for the proper intracellular trafficking of nerve growth factor (NGF), brain-derived neurotrophic factor (BDNF) and neurotensin (NTS) [49,50]. At the interval of one year after identifying PGRN/SORT1 interaction, it was observed that PGRN could interact with tumour necrosis factor receptor 1 (TNFR1) and 2 (TNFR2), acting as a natural-occurring endogenous competitive antagonist of tumour necrosis factor alpha (TNFα) [51]. This observation was, in this case, described as the most interesting and unexpected cornerstone concept of PGRN-related signalling pathway functions and PGRN-associated anti-inflammatory activities. According to the performed kinetic studies using surface plasmon resonance (SPR) analysis, it was observed that PGRN binds to TNFR1 with an affinity comparable to TNFα and binds to TNFR2 with a ~600-fold higher affinity than TNFα itself in a dose-dependent manner [51,52]. As was shown, the interaction between PGRN and TNFR1 and TNFR2 is mediated through the F-A-C domain and their adjacent associated spacer/linker sequences, such as P3, P4 and P5 [53]. Intracellular TNFα-mediated the downstream activation/suppression of signals via TNFR1, and TNFR2 is associated with the competitive counteraction of PGRN exerted through numerous different secondary messengers and transcription factors, such as nuclear factor kappa-light-chain-enhancer of activated B cells (NF-κB), p38 mitogen-activated protein kinase (p38/MAPK), p44/42 mitogen-activated protein kinase (p44/42MAPK), c-Jun N-terminal kinase (JNK), extracellular signal-regulated kinase 1/2 (ERK1/2), protein kinase B (AKT), transcription factor jun-B (jun-B), phosphatidylinositol-4,5-bisphosphate 3-kinase (PI3K) and focal adhesion kinase (FAK) [54,55,56,57]. TNFR1 signalling triggers inflammatory and catabolic (apoptosis) pathways, whereas TNFR2 signalling is associated with the anti-inflammatory and anabolic pathway (survival), whereas those effects are dependent on bilateral outweigh [57]. The activation of transcription factors and MAPK-associated factors with PGRN, TNFR1 and TNFR2 is related to a potentially wide range of functions, such as mRNA transcription for approximately ~2000 TNFα-inducible genes, including cytokines, chemokines, growth factors and proteinases involved in the regulation of the cell cycle, cytoskeletal rearrangement, migration, apoptosis, and survival [53,57]. Another receptor that covers members of the tumour necrosis factor receptor superfamily (TNFRSF) that interacts with PGRN is tumour necrosis factor receptor superfamily member 25 (TNFRSF25), alternatively known as death receptor 3 (DR3) [58]. Prior to the discovery of PGRN, it was established that TNF-like ligand 1A (TL1A) was considered the TNFRSF25 ligand [59]. TL1A is a type II transmembrane protein that can occur in a soluble form via alternative splicing or proteolytic cleavage [60]. The TL1A/TNFRSF25 signalling axis is involved in the pathophysiology of various autoimmune and inflammatory diseases [61]. PGRN could presumably abolish the binding of TL1A to TNFRSF25, as demonstrated in vitro and in vivo [58]. Next, the functional signalling receptor for PGRN covers the ephrin type-A receptor 2 (EPHA2) [62]. It was observed that the binding affinity between PGRN and EPHA2 is closely related to the interaction of PGRN and SORT1 in both solid phase and solution. A particularly high level of expression of EPHA2 is present at the neroanatomical structures, maintaining multiple aspects of synaptic and higher brain functions [63,64]. EPHA2 has also been implicated in promoting many types of cancer, such as breast, prostate, urinary bladder, skin, lung, ovary, and brain cancer [65]. It was suggested that prolonged PGRN/EPHA2 interaction is responsible for downstream signalling by MAPK and AKT, resulting in the stimulation of capillary morphogenesis [66]. This phenomenon could be potentially associated with PGRN overexpression across a broad spectrum of cancers. Recently, PGRN was found to be an essential secreted cofactor that activates and potentiates Toll-like receptor 9 (TLR9)-driven responses [67]. TLR9 is an innate immune receptor responsible for the recognition of the unmethylated CpG-DNA of bacterial, viral, and parasitic origin, as well as self-DNA in immune complexes [68]. Other ligands of TLR9 cover B/K-type and A/D-type of CpG oligodeoxynucleotides (CpG-ODN), which are responsible for T and B cell stimulation and dendritic cell (DC) maturation [69].

## 3. Neurobiology and Executive Functional Aspects of PGRN

Human and preclinical transgenic rodent studies performed postmortem using a complex set of proteomic, transcriptomic and immunofluorescence methods yielded insight into the relevant pattern of PGRN gene expression and its consecutive protein product induction on the sub- and cellular levels within neural components [19,20]. Thus, during ontomorphogenesis, PGRN is widespread and spatiotemporally expressed throughout embryonic, postnatal and adulthood counterparts at multiple and various levels within the cells of the nervous system and its progenitors in a rostral to caudal direction [19,20]. The evaluation of mature specific neuronal subsets indicates that PGRN is constitutively expressed and detected within neurons (NeuN+) and resting microglia (Iba-1+), whereas immunohybridization signals are less present within astrocytes (GFAP+), oligodendrocytes (OLIG2+) and ependymal cells [70]. Furthermore, PGRN gene products are commonly present throughout the brains of mice (C57BL/6) at embryonic day 13.5 (E13.5), gradually increasing by late embryogenesis (E15.5–E18.5) and into early postnatal development (P0–P7), and peaking overall in mature individuals [70]. In the adult brain, PGRN is prominently expressed within all six layers of the neocortex, remaining prominent in the V/VI layers comprising pyramidal and multiform neurons [20,70]. Medium PGRN immunoreactivity is observed within the telencephalon, including the olfactory bulbs (rhinencephalon), striatum, amygdala, and hippocampal formation, demonstrating the highest concentration in CA1, CA2, subiculum and denate gyrus [20,70,71]. An analogous immunoreactivity level is detected in diencephalon, including thalamus, hypothalamus and pituitary gland, mesencephalon, including the reticular nucleus, superior colliculus, and substantia nigra; metencephalon, including the cerebellum, with expression limited to the Purkinje cell layer, as well as myelencephalon [20,70,71]. Differences in the expression of PGRN mRNA and its protein products are also correlated with age, where its immunoreactivity is significantly decreased between 7-week, 20-week and 50-week-old rodents when considering brain parenchyma [72]. In addition, the qualitative ultrastructural analyses of intrinsic and extrinsic nerve elements indicated that PGRN is localized within the secretory pathway, including the endoplasmic reticulum, Golgi apparatus and dense core vesicles, where it is co-transported with BDNF in both antero- and retrograde directions along axons and dendrites [73]. PGRN spatiotemporal secretion and trafficking to intra- and extrasynaptic sites is regulated in a neuronal activity-dependent manner and in response to cellular stressors [73,74]. PGRN has distinct roles that likely contribute to neuronal survival and function, synapse formation, lysosomal and autophagy function, as well as astrogliosis, neurodegeneration and neuroinflammation. Emerging from these studies is the notion that PGRN interacts with multiple receptors to carry out these activities, but a precise description of the mechanisms of action and signalling cascades still remains a research and scientific challenge.

## 4. Role and Function of PGRN in Oncogenesis and Cancer Development

To date, the role of PGRN in the pathogenesis of cancer has been the subject of a number of different studies and has been well established [75]. Nevertheless, the precise delineation of the molecular and cellular mechanisms of PGRN still poses a research challenge. Cancer cells are mainly characterized by rapid proliferation that is inextricably linked with tumour invasion and migration [76]. Regarding the current evidence, the PGRN-mediated regulation of cancer cell proliferation is mediated through several signalling pathways, such as PI3K/AKT, protein kinase C (PKC), c-Myc, ERK1/2 and another MAPK, where some data support the view that PGRN modulates the activity of cyclin-dependent kinase 4 (CKD4) and levels of cyclin B and cyclin D1 [77,78,79]. Recently, it was observed that PGRN levels could modulate transforming growth factor beta (TGF-β) activity through the AKT/mammalian target of the rapamycin kinase (mTOR) pathway, potentially affecting cell proliferation [80]. Another mechanism regarding the actions of PGRN covers the formation of protein tyrosine kinase 2 (PTK2) and paxillin (PXN) complex through ERK1/2 activation that promotes cell invasion and migration, as observed in bladder cancer and mesothelioma [56,81]. In these models, PGRN seems to act with drebrin, constituting an F-acting-binding protein promoting the previously described phenomenon of migration and invasion [82]. Collectively, the role of PGRN in promoting cancer cell proliferation seems to be significant and worthy of further study. Another pathomechanism that involves PGRN action constitutes the resistance to apoptosis as well as the maintenance of cancer stem cells and the tumour microenvironment. It was observed that anti-PGRN antibodies can induce ovarian cancer cell apoptosis through the regulation of cleaved caspase-3 (CASP3), nuclear condensation, DNA fragmentation and poly (ADP-ribose) polymerase (PARP) cleavage [83]. Additional observations suggest that the knockdown of PGRN promotes apoptosis by increasing the ratio of B-cell lymphoma 2 (BCL-2) proteins in cholangiocarcinoma [77]. Cancer stem cells constitute a stem-like cell subpopulation capable of forming tumours in vivo that determine disease recurrence [84]. According to these phenomena, it was observed that PGRN could promote cancer stem cell proliferation in a SORT1-dependent manner in breast cancer [85]. The tumour microenvironment is characterized by breaking away from the original location by crossing the surrounding extracellular matrix (ECM), invading other tissues and organs of the body via direct extension or through the blood or lymphatic system [86]. The breakdown of the ECM and then migration and invasion is often possible via the synthesis of matrix-degrading enzymes like matrix metalloproteinases (MMP) [87]. It was observed that overexpression of PGRN is associated with the increased expression of several MMPs [88]. It was also noticed that PGRN could increase capacity in terms of the invasiveness and metastasis of breast and ovarian cancer via the upregulation of MMP-9 and the activation of matrix metalloproteinase 2 (MMP-2) [36,89]. Vasculo- and angiogenesis constitute the transient processes that occur physiologically in several conditions at times, where these prolonged phenomena are observed during tumourigenesis [90]. In this case, angiogenesis is an essential feature in terms of tumour progression and is consistently related to adverse prognosis. PGRN is observed to be constitutively expressed at low levels in endothelial cells in a quiescent state, whereas it is upregulated regarding the activation of endothelial cells involved with physiological tissue repair, wound healing, the invasion of trophoblasts and placentation [91]. PGRN overexpression was associated with elevated levels of vascular endothelial growth factor (VEGF) through AKT and ERK1/2, contributing to the proliferation of colorectal cancer, breast cancer and oesophageal squamous cell carcinoma cells [92,93]. Other preclinical studies have also indicated that recombinant PGRN increased the expression of VEGF in human umbilical vein endothelial cells (HUVEC) [94]. In this case, PGRN levels correlate with VEGF levels, the size of blood vessels and microvessel density in various cancer models [95]. Recently, PGRN was linked to lymphangiogenesis through the co-expression of VEGF in oesophageal cancer [96]. Other phenomena regarding the role of PGRN in cancer pathogenesis are associated with host immune surveillance, where its expressions seem to be associated with rendering cancer cells less immunogenic and contributing to tumour immune evasion [97]. It was observed that the presence of PGRN could produce cancer cells resistant to natural killer (NK) cytotoxicity through the downregulation of MHC class I chain-related molecule A (MICA) as well as promotion of human leukocyte antigen E (HLA-E), NK group 2D receptor (NKG2D) and NK group 2A receptor (NKG2A) in hepatocellular carcinoma [98]. In the melanoma tumour model B16, it was observed that PGRN could affect tumour proliferation through reduced NK activity and recruitment [99]. Another mechanism observed in the pathogenesis of breast cancer could cover the M2 polarization of macrophages that contributes to CD8+ exclusion [100]. Elevated PGRN levels in pancreatic ductal carcinoma are related to MHC class I expression as well as the depletion of CD8+ lymphocyte infiltration [101].

## 5. Integrative Survey of the Role of PGRN in the Pathogenesis of GBM

**Figure 3:**
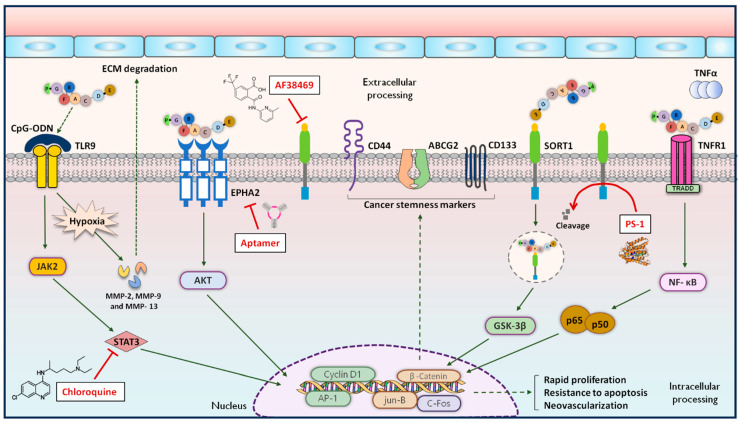
The schematic representation of the physiological and pathological role of progranulin (PGRN) and its associated signalling pathways in the pathogenesis of glioblastoma multiforme (GBM).

The properties of PGRN and its related signalling pathways are currently stay the focus of research on various pre- and clinical models to present its potential role in the implementation of brain tumour therapies. Consecutively, the analysis of available data in the relevant literature exhibits the scarcity of studies regarding the direct subject of astroglial tumours. Regarding the described kinetics of the changes in PGRN in both neuronal and astroglial tissue, as well as many types of cancers, it becomes obvious that this growth factor and its associated signalling axes should be regarded as a new promising research target in the context of GBM (Figure 3). The first insights into how the PGRN is expressed in GBM were provided by Liau et al., which methodically covered the isolation of tumour-specific antibodies and their evaluation in multiple different human gliomas via cDNA microarray hybridization [26]. In this case, the analysis reported that the highest expression of PGRN (~3–30 fold) occurred in GBM compared with a normal brain. Additionally, the administration of synthetic PGRN to primary Fischer 344 (F344) rat astrocytes, as well as three different early passage human GBM cultures, was associated with increased cell proliferation in vitro, whereas the administration of PGRN antibody was associated with the inhibition of cell growth in a dose-dependent pattern. The study of Wang et al. covering analysis of astrocytoma samples with different WHO grades and normal brain tissues using immunohistochemistry (IHC), semi-quantitative real-time PCR (RT-PCR), Western blot and enzyme immunometric assay (ELISA) encompassed the following aspects concerning the significance of GBM [102]. In this case, PGRN expression was little detectable in the normal brain samples but increased in both astrocytoma cells and tumour blood vessels with pathological grading. The immunoreactivity of PGRN in GBM was mainly associated with the smaller fusiform and stellate-shaped tumour cells as well as multi-nucleated giant cells. The blood serum in GBM patients contained significantly higher PGRN levels than the healthy controls. The association between patient survival with GBM was independently associated with PGRN expression and vascular PGRN expression in multivariate analysis. The significant associations between PGRN expression in GBM patients and age, gender, Karnofsky score, tumour location and image status in this population were not observed. The evaluation of the expression profile of PGRN in various brain tumour cell lines, including glioma (H4), GBM (U87, GBM8904, S1R1, PT3) and neuroblastoma (Daoy), was performed in a comprehensive study conducted by Bandey et al. [103]. In this case, the relatively high expression of PGRN was observed within all cell lines except for the PT3 cell line. Moreover, the additional examination of a commercial GBM tissue array showed similar PGRN overexpression results. In the same study, the role of PGRN was evaluated using sh-PGRN constructs in S1R1 cells, where the overexpression of PGRN increased the growth of this cell line. The colony formation ability of GBM cells could also be linked with the presence of PGRN. In this case, the analysis in the clonogenic assay indicated that the PGRN overexpression in the S1R1 cell line is associated with an increase in the colony formation ratio of ~20%, where PGRN knockdown impaired this ability by ~70%. In the next part of the study, the authors analysed the cancer stemness phenomena that cover one of the factors associated with resistance to radio- and chemotherapy. In this case, the subpopulation S1R1 cells with the co-expression of prominin 1 (CD133) was reduced via PGRN knockdown and increased to ~60% via PGRN overexpression. Unquestionably, CD133 constitutes one of the most important cellular markers that covers the glioma stemness marker. Moreover, the levels of expression of other glioma stemness markers like ATP-binding cassette superfamily G member 2 (ABCG2) and CD44 were also increased with PGRN overexpression and decreased with PGRN knockdown, clearly indicating the role of PGRN in regulating the stemness of GBM. Furthermore, it was observed that PGRN affected the functioning of stem-like cells, where PGRN knockdown attenuated the self-renewal potential of this cell population regarding the S1R1 cell line. Additionally, the differentiation process of stem-like cells was also regulated by the presence of PGRN. PGRN knockdown significantly lowers the percentage of GFAP+ and MAP2+ cell subpopulations, indicating that PGRN could be potentially involved in the regulation of neuronal and astrocytic GBM phenotypes. The complex mechanisms for regulating the transcriptional activity of GBM cells are mediated through activating protein 1 (AP-1), which seem to be possible culprits for GBM plasticity and aggressive phenotype transformation. Thus, in the S1R1 cell line, PGRN overexpression elevated, and PGRN knockdown lowered the expression of AP-1 components such as cFos and Jun-B transcripts. Moreover, in the H4 cell line, PGRN expression was positively associated with the expression of transcripts of FosB, Jun-B and c-Fos. Additionally, S1R1 cells with expression of PGRN, c-Fos and JunB also present the increased expression of CD133. In the study performed by Vachher et al., the expression and prognostic values of 11 consecutive members of the adipokines family, including PGRN in low-grade gliomas (LGGs) and GBM, were analysed using gene expression profiling interactive analysis (GEPIA) as well as the Xena server [104]. According to this large-scale analysis, out of 11 adipokines, the mRNA levels of PGRN were significantly upregulated in both LGG and GBM. Moreover, the expression of PGRN was associated with reduced overall survival and disease-free survival for patients with higher mRNA expression in LGGs. In this case, the expression of PGRN showed the worst overall survival of all 11 analysed adipokines in GBM patients. Regarding clinicopathological analysis, PGRN expression was significantly associated between WHO grades II and III, as well as with the histological type in LGG patients. In summary, the authors have proposed PGRN as a potential diagnostic and prognostic markers in the development and progression of gliomas. Regrettably, the lack of studies conducted so far makes it difficult to precisely determine the details of the functional and integrative role of PGRN in the pathogenic mechanisms of GBM. However, the available data raise the possibility of linking the PGRN as a new promising research target in the context of GBM.

## 6. PGRN-Related Receptors and Signalling Pathways in the Context of GBM

In line with actual studies regarding the direct role of PGRN in the pathogenesis of GBM, data concerning related receptors and signalling pathways also offer valuable and relevant enrichment of the discussed insights. The analysis of the properties of PGRN-related receptors and signalling pathways also poses promising research directions for further implementation in the therapy of brain tumours. The available data indicated that SORT1 is also highly upregulated within the cytoplasm and nuclei of gliomas obtained intraoperatively, where its expression level is positively correlated with the tumour grade [105,106]. Further studies also showed that SORT1 expression indicated a significantly poor prognosis, where 2-year survival rates for patients with high expression and low expression levels of SORT1 were 27% and 76%, and the 5-year survival rates of these patients were estimated to be 13% and 51%, respectively [107]. Additional analysis covering U87 and A172 cell lines revealed high levels of SORT1, with a majority distribution in the cytoplasm and a smaller expression in the membrane. In this case, the expression of SORT1 was associated with epithelial–mesenchymal transition (EMT), migration and invasion in GBM cell lines through the activation of the glycogen synthase kinase 3 beta (GSK-3β)/β-catenin signalling pathway. The partial reversibility of those effects was observed after the addition of orally bioavailable small molecule AF38469, which constitutes the potent selective SORT1 inhibitor. Recently, it was observed that presenilin-1 (PS-1) could repress the migration, invasion and EMT of GBM cells through the cleavage of the SORT1 transmembrane domain [108]. This cellular mechanism seems to cover the transduction of the anti-invasive function of PS-1 through the β-catenin signalling pathway and SORT1. The expression of TNFR1 and TNFR2 in a variety of GBM cell lines (e.g., LN-235, LN-319, LN-382, LN-427, LN-443 and HUG-1/2/3), as well as its pathophysiological role, is well-known and well-characterized [109]. It was observed that increased plasma concentrations levels of soluble TNFR1 (sTNFR1) in patients with GBM are associated with shorter survival, independent of age or steroid treatment. The presence of TNFR1-associated death domain (TRADD) plays, in this case, an essential role in the intracellular activation of NF-κB, promoting survival as a key pathooncogenic mechanism and signalling pathway in GBM [110]. Therefore, the overexpression of TNFR1, sTNFR1, as well as TRADD in GBM is significantly associated with a worse prognosis and reduced sensitivity to temozolomide [111]. On the other hand, studies with GBM did not show clear correlations between soluble TNFR2 (sTNFR2) levels and an increased risk and prognosis of this tumour [112]. The available evidence regarding the role of TNFRSF25 as a PGRN receptor in GBM is limited, and its precise functions remain to be clarified. Next, the functional signalling receptor for PGRN covers EPHA2, which is mainly overexpressed in advanced grades of brain tumours, such as GBM [113]. Furthermore, the high expression of EPHA2 has been observed and confirmed within various types of GBM cell lines (e.g., LN-229, T98G, DBTRG-05M, U251MG, BTCOE 4795 and U87-MG) [114,115]. It was observed that the overexpression of EPHA2 is associated with low survival rates and tumour recurrence [115]. At the cellular level, the deregulated expression of EPHA2 was linked to the promotion of tumour aggressiveness, invasion, and metastasis [116]. One of the hallmarks of tumour invasion and aggressiveness associated with GBM development is a high level of angiogenesis [117]. The increased expression of EPHA2, along with other pro-angiogenic molecules, such as VEGF, was found across the microvasculature of GBM, indicating its role in tumour neovascularisation [118]. Furthermore, EPHA2 in GBM seems to regulate the expression of epidermal growth factor receptor (EGFR) and vascular endothelial growth factor receptor 2 (VEGFR-2), which constitute the initial biomarkers of the development of endothelial cells in the neovascularisation process [119]. The significant new properties of nucleic acid-based aptamer have also provided new insights regarding the role of EPHA2 in the pathogenesis of GBM, where targeting EPHA2 by two aptamer agents, such as 40L and its truncated form A40s, resulted in the inhibition of cell growth and the migration of GBM stem-like cells [120]. To date, EPHA2-targeted therapy options in GBM include preclinical, experimental immunotherapy with EPHA2-specific T cells and infusion with ephrin A1 (EFNA1)-based bacterial cytotoxin targeted to EPHA2 [121,122]. Recently, it was observed that PGRN could act as a reinforcing agent for the combination of CpG-ODN and TLR9, which heavily promotes CpG-ODN delivery to the localization of TLR9 [123]. The expression of TLR9 was reported in human, murine and rat GBM cell lines (U251, U87 and C6), as well as in isolated GBM stem-like cells [123,124,125]. TLR9 seems to play a dual role in the pathogenesis of GBM, both as an immune factor eliminating the tumour and as a pro-tumoural molecule [123]. It was observed that the expression level of TLR9 constitutes an independent predictor of survival for the diagnosis of GBM, as well as a prognostic biomarker at an advanced pathological grade [126]. The activation of TLR9 in GBM seems to promote hypoxia-induced tumour cell invasion, probably due to the CpG-ODN effect and the activation of MMP-2, MMP-9 and collagenase 3 (MMP-13), as shown in U373 and U87 cell lines [127,128,129]. The development of GBM stem-like cells is similarly associated with the action of CpG-ODN through Frizzled-4 (Fz-4)/janus kinase 2 (JAK2)/signal transducer and the activator of transcription 3 (STAT3) axis activation [130]. The above-mentioned actions could be abolished through the inhibition of the TLR9 signalling pathway by chloroquine [128,131]. To date, TLR9-targeted therapy options in GBM potentially cover the administration of CpG-28, CpG-ODN-1668, CpG-ODN-107, CpG-1826, CpG-1826 and CpG-ODN without or with various chemotherapeutic agents [123].

## 7. Discussion

**Figure 4:**
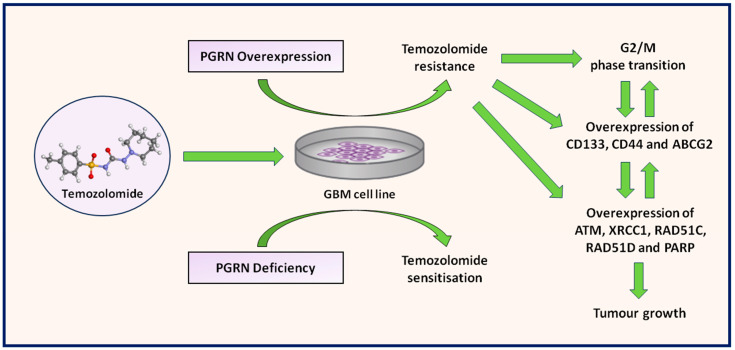
The schematic representation of the possible mechanism of progranulin (PGRN) involvement in temozolomide resistance in glioblastoma multiforme (GBM).

An analysis of the literature shows that the possibility of linking PGRN with various parameters and factors related to GBM seems particularly promising in the clinical context. Temozolomide constitutes a neuropharmacological and anti-cancer drug agent that is currently used together with radiotherapy as part of the first-line treatment of high-grade gliomas [132]. It is an analogue of dacarbazine, where its biochemical structure leads to a lipophilic prodrug with a molecular weight of 194.154 g/mol that functions as a potent alkylating agent [133]. In this case, the precise pharmacodynamic effect of temozolomide covers O^6^-position methylation of guanine (meG) that triggers guanine/thymine mismatch during replication, leading to single-strand deterioration in the genomic DNA of a tumour cell, ultimately resulting in cell cycle arrest in the G2/M phase and apoptosis [134]. The main advantage of this molecule relates to its high penetration capacity in nervous tissue, where its oral bioavailability is practically ~100% [135]. In the clinical context, the use of temozolomide provides an increase in 2-year survival rates (10–25%) as well as 4- and 5-year survival rates, with improvements in terms of progression-free survival time and HRQoL [136,137]. In the last 10 years, the combined use of temozolomide with other cancer drugs has been growing in clinical trials, whereas today, it has yielded survival benefits of only a few months despite promising and encouraging results [138]. To date, the interactions between PGRN and temozolomide were revealed as novel mechanisms that provided insights into drug resistance in GBM therapy (Figure 4). Inherent and acquired temozolomide resistance constitutes a substantial and meaningful obstacle that should be overcome for the successful treatment of GBM [139]. In this case, regarding the highly heterogeneous and mutation-prone nature of this tumour, the resistance ratio to temozolomide is high, covering over ~50% of all GBM patients, which ultimately do not respond to this therapy [140,141]. Another finding highlighted in the study of Bandey et al. was associated with the role of PGRN regarding the temozolomide resistance of GBM [103]. It was observed that PGRN overexpression in S1R1 and H4 cell lines was associated with temozolomide resistance, where PGRN knockdown sensitized these cell lines to temozolomide. Collectively, the presence of PGRN was related to increased DNA synthesis, which directly affected cell growth and temozolomide genotoxicity. Moreover, PGRN-overexpressing S1R1 cells showed the ability to overcome temozolomide-induced G2/M phase arrest, where PGRN-depleted cells were unable to recover from temozolomide-induced G2/M phase arrest, clearly indicating the role of PGRN in maintaining tumour cell cycle integrity. Furthermore, temozolomide-exposed cells from the S1R1 line showed an impaired ability to form a colony by ~40% and decreased its size by ~50%. In this case, concomitant PGRN deficiency was associated with a ~90% decreased ability to form a colony and a ~75% reduction in its size. Another analysis in this study covered an examination of the continuously cultured S1R1 cell at a high dose of temozolomide for 30 days. In this case, the isolated subclones expressed higher levels of PGRN, as well as stemness markers, including CD133, CD44 and ABCG2, than paternal cells. In this regard, PGRN was also denoted as a determinant for GBM cell stemness properties, which could account for its activities in terms of temozolomide resistance. Furthermore, temozolomide resistance in GBM is also associated with the action of DNA repair mechanisms [142]. An important enzyme with a frequent occurrence (35–50%) in GBM cells is O^6^-meG DNA methyltransferase (MGMT) [143]. The function of MGMT is crucial for genome stability via the repair of mutagenic DNA lesion O^6^-meG back to guanine, preventing errors and mismatch during the replication and transcription of DNA [144]. Regarding the observations in the S1R1 cell line, PGRN knockdown was not associated with the changes in MGMT level [103]. The positive correlation between transcripts of DNA repair genes, such as ataxia telangiectasia0mutated kinase (ATM), X-ray repair cross-complementing 1 (XRCC1), RAD51 homolog C (RAD51C), RAD51 homolog D (RAD51D) and PARP was observed in the S1R1 cell line, indicating that PGRN could influence temozolomide toxicity via an MGMT-independent process. The increased expression of this gene group was also observed with the presence of temozolomide and also depleted in the PGRN knockdown S1R1 cell line. Supplementary, the analyses in this study covered a series of orthotopic xenograft implantation experiments in a NOD/SCID mouse model covering the stereotactic injection of S1R1 cells into the striatum. In this case, the injected S1R1 cells were capable of forming tumours within mouse brains, whereas additional temozolomide administration mildly decreased its size. Considerably different, PGRN-depleted cells possess no ability to develop detectable tumours with or without temozolomide treatment according to magnetic resonance imaging (MRI). Another drug agent established as a standard of care during the treatment of GBM is dexamethasone, which is broadly used during the entire course of the disease regarding pre- and postoperative management as well as chemo- and radiation therapy [145]. The main grounds in terms of usage include reduced tumour-associated vasogenic oedema and prophylaxis or the treatment of increased intracranial pressure (ICP) [146]. Another pharmacological effect of dexamethasone includes the reduced dispersal of immortalized and primary GBM cells [147]. To date, no studies have been published regarding PGRN in the context of its potential resistance or susceptibility to dexamethasone therapy in GBM. However, studies focusing on the human multiple myeloma cell line (ARP-1) showed some interesting insights regarding this topic [148]. In this case, the overexpression of PGRN rendered the cells refractory to dexamethasone-mediated apoptosis, increased their ability to form colonies, and form tumours in vivo where glucocorticoid receptor expression and function were unchanged. These findings could also suggest that GBM cells could be potentially resistant to dexamethasone in a PGRN-dependent pathomechanism. To date, no literature has been published regarding the PGRN expression levels within GBM tissues that have a direct effect on resistance to radiation therapy. However, it has been observed that microRNA-107 (miR-107) could enhance radiosensitivity by regulating PGRN in prostate cancer (PC-3) cells [149]. Consecutively, in this study, the observed miR-107 was downregulated, and PGRN was shown to be upregulated in response to ionizing radiation. In the opposite situation, where miR-107 was upregulated and PGRN was downregulated, the promotion of sensitivity of PC-3 cells to ionizing radiation was observed. Other available studies showed that PGRN serum concentration levels in patients with oral squamous cell carcinomas (OSCCs) are not associated with susceptibility levels to radiation therapy [150]. These findings could also suggest that PGRN-related pathomechanisms could be involved in tumour resistance to radiation therapy. Despite ongoing research, GBM still poses a notable challenge due to its multidimensional nature, the required interdisciplinary approach and insufficient therapeutic options with practically certain recurrence [151]. Increasingly, more scientific attention has been paid to phenomena regarding the neuroimmune response associated with the role and activity of various inflammatory mediators, such as cytokines and growth factors [152]. Multifunctional and pleiotropic growth factors of PGRN have aroused substantial interest among neuroscience and oncology researchers due to their solid and noteworthy neurotrophic and immunomodulatory potential [153]. The role of the PGRN and its associated signalling pathways, as analysed in this paper, are well known in the context of physiological regulation within the brain microenvironment and oncogenesis [154]. However, to date, the role and function of PGRN have not been widely discussed in relation to GBM pathogenesis. Regarding the available relevant literature, the expression of PGRN and its associated regulators, receptors, as well as secondary transduction proteins undergo active regulation at the transcriptional and post-translational levels in the development and course of GBM. The general observed tendency of PGRN expression in GBM tumour cell lines and samples obtained intraoperatively from patients undergoing craniotomy or biopsy indicates its abnormal presence associated with the overexpression profile. Furthermore, the increased expression of PGRN seems to be associated with an aggressive phenotype of GBM, often co-occurring with the expression of other GBM-related markers. The expression of PGRN remains generally at constant levels in healthy adult tissues, making it particularly attractive for diagnostic targeting. Given the undeniable role of PGRN in the clinical course of GBM, the combined evaluation of PGRN expression level along with typical GBM-associated biomarkers (e.g., IDH mutations, 1p19q deletion or MGMT promoter methylation) could probably provide strong prognostic factors in predicting the clinical outcomes of this tumour type. Recent studies have demonstrated the importance of considering serum molecular biomarkers in the practice of neuroscience due to their non-invasive and cost-effective collection while also offering extensive information about the patient’s clinical condition [155]. According to the available relevant literature, the serum PGRN levels can be measured in order to provide additional data on the diagnosis and prognosis of GBM. In this case, we support the recommendations that PGRN determination could constitute an auxiliary GBM marker that could result in improving immunodiagnostic accuracy. Taken together, the described data suggest that the increased tissue expression level of the majority of PGRN-related receptors (SORT1, TNFR1, EPHA2 and TLR9) is strongly correlated with the aggressive capacity of GBM and poor prognoses for these patients. The determination of the whole PGRN-related diagnostic panel covering the molecule itself and its related receptors could potentially hold an advantage over standard GBM-related diagnostic algorithms. Collectively, these data provide indirect evidence that modulation within PGRN-associated signalling axes may constitute future potential opportunities in terms of the development of adjuvant GBM therapies. Furthermore, drugs agents that could modulate the PGRN-associated signalling axes might be potentially intriguing as immunological therapy for sensitising tumour cells to chemotherapy and radiotherapy. Preclinical research is essential in the development of potential therapeutic agents. Efforts to identify predictive molecular and immunological GBM markers of response are needed to advance tailored therapy. Therefore, a potential anti-PGRN therapy could cover clinical applications of curcumin or its derivative targeting PGRN/AP-1, which may improve the efficacy of the therapy regimen for GBM patients [103]. The additional combination of curcumin with temozolomide has also demonstrated better efficiency in modulating PGRN/AP-1 as a combination of PARP inhibitor (ABT888) and temozolomide against GBM. In parallel, the use of PGRN-neutralizing antibodies, such as AG01, was associated with the reduced proliferation and migration of triple-negative breast cancer (TNBC) cells [78]. According to these results, it seems that the possible addition of PGRN inhibitors to the chemotherapy regimen of GBM patients can lead to an increased response to treatment. SORT1-targeted agents, such as AF38469 and PS-1, remain, in this case, an attractive therapeutic target, and additional preclinical and clinical studies are still warranted in order to evaluate the clinical activity and benefit of these types of compounds. However, currently, SORT1-targeting agents have recently entered a clinical trial phase regarding thyroid, breast, and ovarian cancer patient populations, where the effects of this adjuvant therapy have been shown to enhance the effect of existing chemotherapy, permitting the targeted entry of a peptide conjugated to docetaxel (TH1902) [156,157,158,159]. The potential adjuvant application of TNFα-inhibitors by afatinib and pomalidomide was observed to effectively inhibit cell growth in multiple subsets of EGFR-expressing GBM, emphasising that the modulation within the PGRN/TNFR1 signalling axis constitutes a very attractive therapeutic research direction [160,161]. The significant new therapeutic options regarding EPHA2-targeting therapy relate to potentially two aptamer agents, such as 40L and A40s, as well as experimental immunotherapy with EPHA2-specific T cells [120,121,122]. Furthermore, until quite recently the potential use of ephrin type-A receptor 3 (EPHA3)-targeting therapy was evaluated in relation to GBM [162]. In this case, the evaluation covered temozolomide-conjugated gold nanoparticles functionalized with an antibody against the EPHA3 (anti-EPHA3-TMZ@GNP) via intranasal administration bypassing the blood–brain barrier (BBB) in a Sprague Dawley rat orthotopic GBM tumour model [163]. The study results demonstrated that the intranasal administration of anti-EPHA3-TMZ@GNP prolonged the median survival time of animals to 42 days and raised the tumour cell apoptosis ratio. In this case, the potential construction of an analogue molecule bearing anti-EPHA2 could be effective in GBM therapy. Another phase I trial covering an evaluation of EPHA2-specific therapy in GBM covered the locoregional administration of interleukin 13 (IL-13) and ephrin A1 (EFNA1)-based bacterial cytotoxins targeted to interleukin 13 receptor subunit alpha 2 (IL13RA2) and EPHA2 receptors to dogs with GBM [122]. Objective tumour volume reductions of 94% were observed in 50% of cases using 0.012–1.278 μg/mL doses of cytotoxins. The promiscuous TLR9-targeted therapy options in GBM involve the administration of various CpG-ODN without or in combination with chemotherapeutic agents [123]. These studies provide preclinical data fundamental to the translation of potential PGRN-associated multireceptor-targeted therapeutic approaches in GBM to clinical settings. Further studies are also needed in order to better understand the specific pharmacobiological mechanisms of these groups of inhibitors [164]. In particular, the “subpopulation” of GBM patients that may likely benefit must be identified [165]. There are existing open questions about the heterogeneous role of PGRN in the course of GBM that demand answers from the academic community represented by neurosurgeons, oncologists and immunologists in the very near future [166]. Firstly, it must be known whether targeting PGRN and other dependent mediators can be involved in the optimization of diagnostic and treatment protocols for GBM patients and predict the overall survival rate and response to therapy among this patient population. Other concerns are related to the lack of studies evaluating the influence of PGRN and its associated receptors and signalling pathways with resistance to radiotherapy and adjuvant drug agents like steroids.

## 8. Conclusions

An increasing number of studies have shown that PGRN is an important factor in oncogenesis. In this review, we presented some of the important biological and molecular effects of PGRN throughout the course of GBM, as well as several associated diagnostics and therapeutic targets. Collectively, these results provide strong molecular evidence that PGRN constitutes a potential prognostic biomarker for GBM and is an attractive therapeutic target for this tumour therapy. Comprehensive analysis of the role of the PGRN and its associated signalling axes, particularly in the context of GBM and the possibility of its modulation with drugs and immunomodulatory treatments may bring tangible benefits associated with understanding this type of tumour and the possibility of comprehensive influence exerted on the immune response in order to obtain superior clinical results. Future studies are warranted to elucidate the complex role of PGRN, its associated receptors and signalling pathways in GBM.

## References

[1] Wu W., Klockow J.L., Zhang M., Lafortune F., Chang E., Jin L., Wu Y., Daldrup-Link H.E. (2021). Glioblastoma multiforme (GBM): An overview of current therapies and mechanisms of resistance. Pharmacol. Res..

[2] Hanif F., Muzaffar K., Perveen K., Malhi S.M., Simjee S.U. (2017). Glioblastoma Multiforme: A Review of its Epidemiology and Pathogenesis through Clinical Presentation and Treatment. Asian Pac. J. Cancer Prev. APJCP.

[3] Grochans S., Cybulska A.M., Simińska D., Korbecki J., Kojder K., Chlubek D., Baranowska-Bosiacka I. (2022). Epidemiology of Glioblastoma Multiforme–Literature Review. Cancers.

[4] Grech N., Dalli T., Mizzi S., Meilak L., Calleja N., Zrinzo A. (2020). Rising Incidence of Glioblastoma Multiforme in a Well-Defined Population. Cureus.

[5] Olson J.J., Ryken T.C. (2020). Congress of neurological surgeons systematic review and evidence-based clinical practice parameter guidelines for the treatment of adults with newly diagnosed glioblastoma: Introduction and Methods. J. Neuro-Oncol..

[6] Goel N.J., Bird C.E., Hicks W.H., Abdullah K.G. (2021). Economic implications of the modern treatment paradigm of glioblastoma: An analysis of global cost estimates and their utility for cost assessment. J. Med Econ..

[7] Rodríguez-Camacho A., Flores-Vázquez J.G., Moscardini-Martelli J., Torres-Ríos J.A., Olmos-Guzmán A., Ortiz-Arce C.S., Cid-Sánchez D.R., Pérez S.R., Macías-González M.D.S., Hernández-Sánchez L.C. (2022). Glioblastoma Treatment: State-of-the-Art and Future Perspectives. Int. J. Mol. Sci..

[8] Greten F.R., Grivennikov S.I. (2019). Inflammation and Cancer: Triggers, Mechanisms, and Consequences. Immunity.

[9] Roesler R., Dini S.A., Isolan G.R. (2021). Neuroinflammation and immunoregulation in glioblastoma and brain metastases: Recent developments in imaging approaches. Clin. Exp. Immunol..

[10] Brat D.J., Castellano-Sanchez A.A., Hunter S.B., Pecot M., Cohen C., Hammond E.H., Devi S.N., Kaur B., Van Meir E.G. (2004). Pseudopalisades in Glioblastoma Are Hypoxic, Express Extracellular Matrix Proteases, and Are Formed by an Actively Migrating Cell Population. Cancer Res..

[11] DeCordova S., Shastri A., Tsolaki A.G., Yasmin H., Klein L., Singh S.K., Kishore U. (2020). Molecular Heterogeneity and Immunosuppressive Microenvironment in Glioblastoma. Front. Immunol..

[12] Albulescu R., Codrici E., Popescu I.D., Mihai S., Necula L.G., Petrescu D., Teodoru M., Tanase C.P. (2013). Cytokine Patterns in Brain Tumour Progression. Mediat. Inflamm..

[13] Yeo E.C.F., Brown M.P., Gargett T., Ebert L.M. (2021). The Role of Cytokines and Chemokines in Shaping the Immune Microenvironment of Glioblastoma: Implications for Immunotherapy. Cells.

[14] Ventura E., Ducci G., Dominguez R.B., Ruggiero V., Belfiore A., Sacco E., Vanoni M., Iozzo R.V., Giordano A., Morrione A. (2023). Progranulin Oncogenic Network in Solid Tumors. Cancers.

[15] Baker M., Mackenzie I.R., Pickering-Brown S.M., Gass J., Rademakers R., Lindholm C., Snowden J., Adamson J., Sadovnick A.D., Rollinson S. (2006). Mutations in progranulin cause tau-negative frontotemporal dementia linked to chromosome 17. Nature.

[16] Cruts M., Gijselinck I., van der Zee J., Engelborghs S., Wils H., Pirici D., Rademakers R., Vandenberghe R., Dermaut B., Martin J.-J. (2006). Null mutations in progranulin cause ubiquitin-positive frontotemporal dementia linked to chromosome 17q21. Nature.

[17] He Z., Bateman A. (2003). Progranulin (granulin-epithelin precursor, PC-cell-derived growth factor, acrogranin) mediates tissue repair and tumorigenesis. J. Mol. Med..

[18] Liu C., Li J., Shi W., Zhang L., Liu S., Lian Y., Liang S., Wang H. (2020). Progranulin Regulates Inflammation and Tumor. Anti-Inflamm. Anti-Allergy Agents Med. Chem..

[19] Daniel R., He Z., Carmichael K.P., Halper J., Bateman A. (2000). Cellular Localization of Gene Expression for Progranulin. J. Histochem. Cytochem..

[20] Daniel R., Daniels E., He Z., Bateman A. (2003). Progranulin (acrogranin/PC cell-derived growth factor/granulin-epithelin precursor) is expressed in the placenta, epidermis, microvasculature, and brain during murine development. Dev. Dyn..

[21] Bateman A., Bennett H.P.J. (2009). The granulin gene family: From cancer to dementia. BioEssays.

[22] De Muynck L., Van Damme P. (2011). Cellular Effects of Progranulin in Health and Disease. J. Mol. Neurosci..

[23] Koo D.H., Park C.-Y., Lee E.S., Ro J., Oh S.W. (2012). Progranulin as a Prognostic Biomarker for Breast Cancer Recurrence in Patients Who Had Hormone Receptor-Positive Tumors: A Cohort Study. PLoS ONE.

[24] Serrero G. (2021). Progranulin/GP88, A Complex and Multifaceted Player of Tumor Growth by Direct Action and via the Tumor Mi-croenvironment. Adv. Exp. Med. Biol..

[25] Buraschi S., Xu S.-Q., Stefanello M., Moskalev I., Morcavallo A., Genua M., Tanimoto R., Birbe R., Peiper S.C., Gomella L.G. (2016). Suppression of progranulin expression inhibits bladder cancer growth and sensitizes cancer cells to cisplatin. Oncotarget.

[26] Liau L.M., Lallone R.L., Seitz R.S., Buznikov A., Gregg J.P., Kornblum H.I., Nelson S.F., Bronstein J.M. (2000). Identification of a human glio-ma-associated growth factor gene, granulin, using differential immuno-absorption. Cancer Res..

[27] Shoyab M., McDonald V.L., Byles C., Todaro G.J., Plowman G.D. (1990). Epithelins 1 and 2: Isolation and characterization of two cysteine-rich growth-modulating proteins. Proc. Natl. Acad. Sci. USA.

[28] Bateman A., Belcourt D., Bennett H., Lazure C., Solomon S. (1990). Granulins, a novel class of peptide from leukocytes. Biochem. Biophys. Res. Commun..

[29] Bhandari V., Palfree R.G., Bateman A. (1992). Isolation and sequence of the granulin precursor cDNA from human bone marrow reveals tandem cysteine-rich granulin domains. Proc. Natl. Acad. Sci. USA.

[30] Plowman G., Green J., Neubauer M., Buckley S., McDonald V., Todaro G., Shoyab M. (1992). The epithelin precursor encodes two proteins with opposing activities on epithelial cell growth. J. Biol. Chem..

[31] Anakwe O.O., Anakwe O.O., Gerton G.L. (1990). Acrosome biogenesis begins during meiosis: Evidence from the synthesis and distribution of an acrosomal glycoprotein, acrogranin, during guinea pig spermatogenesis. Biol. Reprod..

[32] Baba T., Hoff H.B., Nemoto H., Lee H., Orth J., Arai Y., Gerton G.L. (1993). Acrogranin, an acrosomal cysteine-rich glycoprotein, is the precursor of the growth-modulating peptides, granulins, and epithelins, and is expressed in somatic as well as male germ cells. Mol. Reprod. Dev..

[33] Zhou J., Gao G., Crabb J., Serrero G. (1993). Purification of an autocrine growth factor homologous with mouse epithelin precursor from a highly tumorigenic cell line. J. Biol. Chem..

[34] Zanocco-Marani T., Bateman A., Romano G., Valentinis B., He Z.H., Baserga R. (1999). Biological activities and signaling pathways of the granulin/epithelin precursor. Cancer Res..

[35] Serrero G. (2003). Autocrine growth factor revisited: PC-cell-derived growth factor (progranulin), a critical player in breast cancer tumorigenesis. Biochem. Biophys. Res. Commun..

[36] Tangkeangsirisin W., Serrero G. (2004). PC cell-derived growth factor (PCDGF/GP88, progranulin) stimulates migration, invasiveness and VEGF expression in breast cancer cells. Carcinogenesis.

[37] Parnell P.G., Wunderlich J., Carter B., Halper J. (1990). Purification of transforming growth factor type e. J. Cell. Biochem..

[38] Palfree R.G.E., Bennett H.P.J., Bateman A. (2015). The Evolution of the Secreted Regulatory Protein Progranulin. PLoS ONE.

[39] Bateman A., Bennett H. (1998). Granulins: The structure and function of an emerging family of growth factors. J. Endocrinol..

[40] Hrabal R., Chen Z., James S., Bennett H.P., Ni F. (1996). The hairpin stack fold, a novel protein architecture for a new family of protein growth factors. Nat. Struct. Mol. Biol..

[41] Zhu J., Nathan C., Jin W., Sim D., Ashcroft G.S., Wahl S.M., Lacomis L., Erdjument-Bromage H., Tempst P., Wright C.D. (2002). Conversion of Proepithelin to Epithelins: Roles of SLPI and Elastase in Host Defense and Wound Repair. Cell.

[42] Xu D., Suenaga N., Edelmann M.J., Fridman R., Muschel R.J., Kessler B.M. (2008). Novel MMP-9 Substrates in Cancer Cells Revealed by a Label-free Quantitative Proteomics Approach. Mol. Cell. Proteom..

[43] Suh H.-S., Choi N., Tarassishin L., Lee S.C. (2012). Regulation of Progranulin Expression in Human Microglia and Proteolysis of Progranulin by Matrix Metalloproteinase-12 (MMP-12). PLoS ONE.

[44] Bai X.-H., Wang D.-W., Kong L., Zhang Y., Luan Y., Kobayashi T., Kronenberg H.M., Yu X.-P., Liu C.-J. (2009). ADAMTS-7, a Direct Target of PTHrP, Adversely Regulates Endochondral Bone Growth by Associating with and Inactivating GEP Growth Factor. Mol. Cell. Biol..

[45] Kessenbrock K., Fröhlich L., Sixt M., Lämmermann T., Pfister H., Bateman A., Belaaouaj A., Ring J., Ollert M., Fässler R. (2008). Proteinase 3 and neutrophil elastase enhance inflammation in mice by inactivating antiinflammatory progranulin. J. Clin. Investig..

[46] Devoogdt N., Rasool N., Hoskins E., Simpkins F., Tchabo N., Kohn E.C. (2009). Overexpression of protease inhibitor-dead secretory leukocyte protease inhibitor causes more aggressive ovarian cancer *in vitro* and *in vivo*. Cancer Sci..

[47] Okura H., Yamashita S., Ohama T., Saga A., Yamamoto-Kakuta A., Hamada Y., Sougawa N., Ohyama R., Sawa Y., Matsuyama A. (2010). HDL/Apolipoprotein A-I Binds to Macrophage-Derived Progranulin and Suppresses its Conversion into Proinflammatory Granulins. J. Atheroscler. Thromb..

[48] Willnow T.E., Petersen C.M., Nykjaer A. (2008). VPS10P-domain receptors—Regulators of neuronal viability and function. Nat. Rev. Neurosci..

[49] Chen Z.-Y., Ieraci A., Teng H., Dall H., Meng C.-X., Herrera D.G., Nykjaer A., Hempstead B.L., Lee F.S. (2005). Sortilin Controls Intracellular Sorting of Brain-Derived Neurotrophic Factor to the Regulated Secretory Pathway. J. Neurosci..

[50] Nielsen M.S., Jacobsen C., Olivecrona G., Gliemann J., Petersen C.M. (1999). Sortilin/Neurotensin Receptor-3 Binds and Mediates Degradation of Lipoprotein Lipase. J. Biol. Chem..

[51] Tang W., Lu Y., Tian Q.-Y., Zhang Y., Guo F.-J., Liu G.-Y., Syed N.M., Lai Y., Lin E.A., Kong L. (2011). The Growth Factor Progranulin Binds to TNF Receptors and Is Therapeutic Against Inflammatory Arthritis in Mice. Science.

[52] Liu C.-J. (2011). Progranulin: A promising therapeutic target for rheumatoid arthritis. FEBS Lett..

[53] Tian Q., Zhao Y., Mundra J.J., Gonzalez-Gugel E., Jian J., Uddin S.M.Z., Liu C. (2014). Three TNFR-binding domains of PGRN act independently in inhibition of TNF-alpha binding and activity. Front. Biosci..

[54] Feng J.Q., Guo F., Jiang B., Zhang Y., Frenkel S., Wang D., Tang W., Xie Y., Liu C. (2010). Granulin epithelin precursor: A bone morphogenic protein 2-inducible growth factor that activates Erk1/2 signaling and JunB transcription factor in chondrogenesis. FASEB J..

[55] Hwang H.-J., Jung T.W., Hong H.C., Choi H.Y., Seo J.-A., Kim S.G., Kim N.H., Choi K.M., Choi D.S., Baik S.H. (2013). Progranulin Protects Vascular Endothelium against Atherosclerotic Inflammatory Reaction via Akt/eNOS and Nuclear Factor-κB Pathways. PLoS ONE.

[56] Monami G., Gonzalez E.M., Hellman M., Gomella L.G., Baffa R., Iozzo R.V., Morrione A. (2006). Proepithelin Promotes Migration and Invasion of 5637 Bladder Cancer Cells through the Activation of ERK1/2 and the Formation of a Paxillin/FAK/ERK Complex. Cancer Res..

[57] Wang B.C., Liu H., Talwar A., Jian J. (2015). New discovery rarely runs smooth: An update on progranulin/TNFR interactions. Protein Cell.

[58] Liu C., Li X.-X., Gao W., Liu W., Liu D.-S. (2014). Progranulin-Derived Atsttrin Directly Binds to TNFRSF25 (DR3) and Inhibits TNF-Like Ligand 1A (TL1A) Activity. PLoS ONE.

[59] Bittner S., Ehrenschwender M. (2017). Multifaceted death receptor 3 signaling—Promoting survival and triggering death. FEBS Lett..

[60] Bittner S., Knoll G., Füllsack S., Kurz M., Wajant H., Ehrenschwender M. (2015). Soluble TL1A is sufficient for activation of death receptor 3. FEBS J..

[61] Aiba Y., Nakamura M. (2013). The Role of TL1A and DR3 in Autoimmune and Inflammatory Diseases. Mediat. Inflamm..

[62] Neill T., Buraschi S., Goyal A., Sharpe C., Natkanski E., Schaefer L., Morrione A., Iozzo R.V. (2016). EphA2 is a functional receptor for the growth factor progranulin. J. Cell Biol..

[63] Yamaguchi Y., Pasquale E.B. (2004). Eph receptors in the adult brain. Curr. Opin. Neurobiol..

[64] Murai K.K., Pasquale E.B. (2004). Eph Receptors, Ephrins, and Synaptic Function. Neuroscientist.

[65] Tandon M., Vemula S.V., Mittal S.K. (2010). Emerging strategies for EphA2 receptor targeting for cancer therapeutics. Expert Opin. Ther. Targets.

[66] Chitramuthu B., Bateman A. (2016). Progranulin and the receptor tyrosine kinase EphA2, partners in crime?. J. Cell Biol..

[67] Park B., Buti L., Lee S., Matsuwaki T., Spooner E., Brinkmann M.M., Nishihara M., Ploegh H.L. (2011). Granulin Is a Soluble Cofactor for Toll-like Receptor 9 Signaling. Immunity.

[68] Vollmer J. (2006). TLR9 in Health and Disease. Int. Rev. Immunol..

[69] Huang X., Yang Y. (2010). Targeting the TLR9–MyD88 pathway in the regulation of adaptive immune responses. Expert Opin. Ther. Targets.

[70] Petkau T.L., Neal S., Orban P., MacDonald J., Hill A., Lu G., Feldman H., Mackenzie I., Leavitt B. (2010). Progranulin expression in the developing and adult murine brain. J. Comp. Neurol..

[71] Bhandari V., Giaid A., Bateman A. (1993). The complementary deoxyribonucleic acid sequence, tissue distribution, and cellular localization of the rat granulin precursor. Endocrinology.

[72] Matsuwaki T., Asakura R., Suzuki M., Yamanouchi K., Nishihara M. (2011). Age-Dependent Changes in Progranulin Expression in the Mouse Brain. J. Reprod. Dev..

[73] Petoukhov E., Fernando S., Mills F., Shivji F., Hunter D., Krieger C., Silverman M.A., Bamji S.X. (2013). Activity-dependent secretion of progranulin from synapses. J. Cell Sci..

[74] Townley R.A., Boeve B.F., Benarroch E.E. (2018). Progranulin. Neurology.

[75] Zhou C., Huang Y., Wu J., Wei Y., Chen X., Lin Z., Nie S. (2021). A narrative review of multiple mechanisms of progranulin in cancer: A potential target for anti-cancer therapy. Transl. Cancer Res..

[76] Ishimoto K., Minami K., Otagaki S., Tsujiuchi T. (2019). Rapid establishment of highly migratory cells from cancer cells for investigating cellular functions. J. Recept. Signal Transduct..

[77] Daya M., Loilome W., Techasen A., Thanee M., Sa-Ngiamwibool P., Titapun A., Yongvanit P., Namwat N. (2018). Progranulin modulates cholangiocarcinoma cell proliferation, apoptosis, and motility via the PI3K/pAkt pathway. OncoTargets Ther..

[78] Guha R., Yue B., Dong J., Banerjee A., Serrero G. (2021). Anti-progranulin/GP88 antibody AG01 inhibits triple negative breast cancer cell proliferation and migration. Breast Cancer Res. Treat..

[79] Arechavaleta-Velasco F., Perez-Juarez C.E., Gerton G.L., Diaz-Cueto L. (2017). Progranulin and its biological effects in cancer. Med. Oncol..

[80] Yabe K., Yamamoto Y., Takemura M., Hara T., Tsurumi H., Serrero G., Nabeshima T., Saito K. (2021). Progranulin depletion inhibits proliferation via the transforming growth factor beta/SMAD family member 2 signaling axis in Kasumi-1 cells. Heliyon.

[81] Ventura E., Xie C., Buraschi S., Belfiore A., Iozzo R.V., Giordano A., Morrione A. (2022). Complexity of progranulin mechanisms of action in mesothelioma. J. Exp. Clin. Cancer Res..

[82] Xu S.-Q., Buraschi S., Morcavallo A., Genua M., Shirao T., Peiper S.C., Gomella L.G., Birbe R., Belfiore A., Iozzo R.V. (2015). A novel role for drebrin in regulating progranulin bioactivity in bladder cancer. Oncotarget.

[83] Kamrava M., Simpkins F., Alejandro E., Michener C., Meltzer E., Kohn E.C. (2005). Lysophosphatidic acid and endothelin-induced proliferation of ovarian cancer cell lines is mitigated by neutralization of granulin–epithelin precursor (GEP), a prosurvival factor for ovarian cancer. Oncogene.

[84] Kuşoğlu A., Avcı B. (2018). Cancer stem cells: A brief review of the current status. Gene.

[85] Berger K., Pauwels E., Parkinson G., Landberg G., Le T., Demillo V.G., Lumangtad L.A., Jones D.E., Islam A., Olsen R. (2021). Reduction of Progranulin-Induced Breast Cancer Stem Cell Propagation by Sortilin-Targeting Cyclotriazadisulfonamide (CADA) Compounds. J. Med. Chem..

[86] Anderson N.M., Simon M.C. (2020). The tumor microenvironment. Curr. Biol..

[87] Niland S., Riscanevo A.X., Eble J.A. (2021). Matrix Metalloproteinases Shape the Tumor Microenvironment in Cancer Progression. Int. J. Mol. Sci..

[88] Lan Y.-J., Sam N.B., Cheng M.-H., Pan H.-F., Gao J. (2021). Progranulin as a Potential Therapeutic Target in Immune-Mediated Diseases. J. Inflamm. Res..

[89] Liu Y., Xi L., Liao G., Wang W., Tian X., Wang B., Chen G., Han Z., Wu M., Wang S. (2007). Inhibition of PC cell-derived growth factor (PCDGF)/granulin-epithelin precursor (GEP) decreased cell proliferation and invasion through downregulation of cyclin D and CDK 4 and inactivation of MMP-2. BMC Cancer.

[90] Viallard C., Larrivée B. (2017). Tumor angiogenesis and vascular normalization: Alternative therapeutic targets. Angiogenesis.

[91] Toh H., Cao M., Daniels E., Bateman A. (2013). Expression of the Growth Factor Progranulin in Endothelial Cells Influences Growth and Development of Blood Vessels: A Novel Mouse Model. PLoS ONE.

[92] Chen X.-Y., Li J.-S., Liang Q.-P., He D.-Z., Zhao J. (2008). Expression of PC cell-derived growth factor and vascular endothelial growth factor in esophageal squamous cell carcinoma and their clinicopathologic significance. Chin. Med. J..

[93] Yang D., Wang L.L., Dong T.T., Shen Y.H., Guo X.S., Liu C.Y., Liu J., Zhang P., Li J., Sun Y.P. (2015). Progranulin promotes colorectal cancer proliferation and angiogenesis through TNFR2/Akt and ERK signaling pathways. Am. J. Cancer Res..

[94] Huang H., Li J., Lu Y., Min L., Li D., Dai L. (2015). Role of midkine-progranulin interaction during angiogenesis of hepatocellular carcinoma. Int. J. Clin. Exp. Pathol..

[95] Binișor I., Baniță I.M., Alexandru D., Mehedinți M.C., Jurja S., Andrei A.-M., Pisoschi C.G. (2021). Progranulin: A proangiogenic factor in visceral adipose tissue in tumoral and non-tumoral visceral pathology. Exp. Ther. Med..

[96] Li G., Dong T., Yang D., Gao A., Luo J., Yang H., Wang L. (2018). Progranulin promotes lymphangiogenesis through VEGF-C and is an independent risk factor in human esophageal cancers. Hum. Pathol..

[97] Kwack K.H., Lee H.-W. (2017). Progranulin Inhibits Human T Lymphocyte Proliferation by Inducing the Formation of Regulatory T Lymphocytes. Mediat. Inflamm..

[98] Cheung P.F., Yip C.W., Wong N.C., Fong D.Y., Ng L.W., Wan A.M., Wong C.K., Cheung T.T., Ng I.O., Poon R.T. (2014). Granulin–Epithelin Precursor Renders Hepatocellular Carcinoma Cells Resistant to Natural Killer Cytotoxicity. Cancer Immunol. Res..

[99] Voshtani R., Song M., Wang H., Li X., Zhang W., Tavallaie M.S., Yan W., Sun J., Wei F., Ma X. (2019). Progranulin promotes melanoma progression by inhibiting natural killer cell recruitment to the tumor microenvironment. Cancer Lett..

[100] Fang W., Zhou T., Shi H., Yao M., Zhang D., Qian H., Zeng Q., Wang Y., Jin F., Chai C. (2021). Progranulin induces immune escape in breast cancer via up-regulating PD-L1 expression on tumor-associated macrophages (TAMs) and promoting CD8+ T cell exclusion. J. Exp. Clin. Cancer Res..

[101] Cheung P.F., Yang J., Fang R., Borgers A., Krengel K., Stoffel A., Althoff K., Yip C.W., Siu E.H.L., Ng L.W.C. (2022). Progranulin mediates immune evasion of pancreatic ductal adenocarcinoma through regulation of MHCI expression. Nat. Commun..

[102] Wang M., Li G., Yin J., Lin T., Zhang J. (2011). Progranulin overexpression predicts overall survival in patients with glioblastoma. Med Oncol..

[103] Bandey I., Chiou S.-H., Huang A.-P., Tsai J.-C., Tu P.-H. (2014). Progranulin promotes Temozolomide resistance of glioblastoma by orchestrating DNA repair and tumor stemness. Oncogene.

[104] Vachher M., Arora K., Burman A., Kumar B. (2019). NAMPT, GRN, and SERPINE1 signature as predictor of disease progression and survival in gliomas. J. Cell. Biochem..

[105] Xiong J., Zhou L., Yang M., Lim Y., Zhu Y.-H., Fu D.-L., Li Z.-W., Zhong J.-H., Xiao Z.-C., Zhou X.-F. (2013). ProBDNF and its receptors are upregulated in glioma and inhibit the growth of glioma cells in vitro. Neuro-Oncol..

[106] Marsland M., Dowdell A., Faulkner S., Gedye C., Lynam J., Griffin C.P., Marsland J., Jiang C.C., Hondermarck H. (2023). The Membrane Protein Sortilin Is a Potential Biomarker and Target for Glioblastoma. Cancers.

[107] Yang W., Wu P.-F., Ma J.-X., Liao M.-J., Wang X.-H., Xu L.-S., Xu M.-H., Yi L. (2019). Sortilin promotes glioblastoma invasion and mesenchymal transition through GSK-3β/β-catenin/twist pathway. Cell Death Dis..

[108] Yang W., Xiang Y., Liao M.-J., Wu P.-F., Yang L., Huang G.-H., Shi B.-Z., Yi L., Lv S.-Q. (2021). Presenilin1 inhibits glioblastoma cell invasiveness via promoting Sortilin cleavage. Cell Commun. Signal..

[109] Kato T., Sawamura Y., Tada M., Sakuma S., Sudo M., Abe H. (1995). p55 and p 75 Tumor Necrosis Factor Receptor Expression on Human Glioblastoma Cells. Neurol. Med.-Chir..

[110] Chakraborty S., Li L., Tang H., Xie Y., Puliyappadamba V.T., Raisanen J., Burma S., Boothman D.A., Cochran B., Wu J. (2013). Cytoplasmic TRADD Confers a Worse Prognosis in Glioblastoma. Neoplasia.

[111] Cahill K.E., Morshed R.A., Yamini B. (2015). Nuclear factor-κB in glioblastoma: Insights into regulators and targeted therapy. Neuro-Oncology.

[112] Kartikasari A.E.R., Cassar E., Razqan M.A.M., Szydzik C., Huertas C.S., Mitchell A., Plebanski M. (2022). Elevation of circulating TNF receptor 2 in cancer: A systematic meta-analysis for its potential as a diagnostic cancer biomarker. Front. Immunol..

[113] Wykosky J., Gibo D.M., Stanton C., Debinski W. (2005). EphA2 as a Novel Molecular Marker and Target in Glioblastoma Multiforme. Mol. Cancer Res..

[114] Ferluga S., Tomé C.M.L., Herpai D.M., D’Agostino R., Debinski W. (2016). Simultaneous targeting of Eph receptors in glioblastoma. Oncotarget.

[115] Baharuddin W.N.A., Yusoff A.A.M., Abdullah J.M., Osman Z.F., Ahmad F. (2018). Roles of EphA2 Receptor in Angiogenesis Signaling Pathway of Glioblastoma Multiforme. Malays. J. Med. Sci..

[116] Miao H., Gale N.W., Guo H., Qian J., Petty A., Kaspar J., Murphy A.J., Valenzuela D.M., Yancopoulos G., Hambardzumyan D. (2014). EphA2 promotes infiltrative invasion of glioma stem cells in vivo through cross-talk with Akt and regulates stem cell properties. Oncogene.

[117] Das S., Marsden P.A. (2013). Angiogenesis in Glioblastoma. New Engl. J. Med..

[118] Shen L., Sun R., Kan S., Wang Z., Yu Z. (2021). EphA2, vascular endothelial growth factor, and vascular endothelial growth factor correlate with adverse outcomes and poor survival in patients with glioma. Medicine.

[119] Guo S., Colbert L.S., Fuller M., Zhang Y., Gonzalez-Perez R.R. (2010). Vascular endothelial growth factor receptor-2 in breast cancer. Biochim. et Biophys. Acta (BBA) Rev. Cancer.

[120] Affinito A., Quintavalle C., Esposito C.L., Roscigno G., Giordano C., Nuzzo S., Ricci-Vitiani L., Scognamiglio I., Minic Z., Pallini R. (2020). Targeting Ephrin Receptor Tyrosine Kinase A2 with a Selective Aptamer for Glioblastoma Stem Cells. Mol. Ther. Nucleic Acids.

[121] Chow K.K., Naik S., Kakarla S., Brawley V.S., Shaffer D.R., Yi Z., Rainusso N., Wu M.-F., Liu H., Kew Y. (2013). T Cells Redirected to EphA2 for the Immunotherapy of Glioblastoma. Mol. Ther..

[122] Rossmeisl J.H., Herpai D., Quigley M., Cecere T.E., Robertson J.L., D’agostino R.B., Hinckley J., Tatter S.B., Dickinson P.J., Debinski W. (2020). Phase I trial of convection-enhanced delivery of IL13RA2 and EPHA2 receptor targeted cytotoxins in dogs with spontaneous intracranial gliomas. Neuro-Oncology.

[123] Fehri E., Ennaifer E., Rhouma R.B.H., Ardhaoui M., Boubaker S. (2022). TLR9 and Glioma: Friends or Foes?. Cells.

[124] Leng L., Jiang T., Zhang Y. (2012). TLR9 expression is associated with prognosis in patients with glioblastoma multiforme. J. Clin. Neurosci..

[125] Meng Y., Kujas M., Marie Y., Paris S., Thillet J., Delattre J.-Y., Carpentier A.F. (2008). Expression of TLR9 within human glioblastoma. J. Neuro-Oncol..

[126] Miyar A., Habibi I., Ebrahimi A., Mansourpour D., Mokarizadeh A., Rajabi A., Farshgar R., Eshaghzadeh M., Zamani-Ahmadmahmudi M., Nodushan S.M.H.T. (2016). Predictive and prognostic value of TLR9 and NFKBIA gene expression as potential biomarkers for human glioma diagnosis. J. Neurol. Sci..

[127] Merrell M.A., Ilvesaro J.M., Lehtonen N., Sorsa T., Gehrs B., Rosenthal E., Chen D., Shackley B., Harris K.W., Selander K.S. (2006). Toll-Like Receptor 9 Agonists Promote Cellular Invasion by Increasing Matrix Metalloproteinase Activity. Mol. Cancer Res..

[128] Wang C., Cao S., Yan Y., Ying Q., Jiang T., Xu K., Wu A. (2010). TLR9 expression in glioma tissues correlated to glioma progression and the prognosis of GBM patients. BMC Cancer.

[129] Sandholm J., Tuomela J., Kauppila J.H., Harris K.W., Graves D., Selander K.S. (2014). Hypoxia regulates Toll-like receptor-9 expression and invasive function in human brain cancer cells in vitro. Oncol. Lett..

[130] Herrmann A., Cherryholmes G., Schroeder A., Phallen J., Alizadeh D., Xin H., Wang T., Lee H., Lahtz C., Swiderski P. (2014). TLR9 Is Critical for Glioma Stem Cell Maintenance and Targeting. Cancer Res.

[131] Chaudhary R., Morris R.J., Steinson E. (2021). The multifactorial roles of microglia and macrophages in the maintenance and progression of glioblastoma. J. Neuroimmunol..

[132] Karachi A., Dastmalchi F., Mitchell D.A., Rahman M. (2018). Temozolomide for immunomodulation in the treatment of glioblastoma. Neuro-Oncology.

[133] Ahad A., Shakeel F., Raish M., Ahmad A., Bin Jardan Y.A., Al-Jenoobi F.I., Al-Mohizea A.M. (2022). Thermodynamic Solubility Profile of Temozolomide in Different Commonly Used Pharmaceutical Solvents. Molecules.

[134] Zhang J., Stevens M.F., Bradshaw T.D. (2012). Temozolomide: Mechanisms of Action, Repair and Resistance. Curr. Mol. Pharmacol..

[135] Friedman H.S., Kerby T., Calvert H. (2000). Temozolomide and treatment of malignant glioma. Clin. Cancer Res..

[136] Sher D.J., Henson J.W., Avutu B., Hochberg F.H., Batchelor T.T., Martuza R.L., Barker F.G., Loeffler J.S., Chakravarti A. (2008). The added value of concurrently administered temozolomide versus adjuvant temozolomide alone in newly diagnosed glioblastoma. J. Neuro-Oncol..

[137] Back M.F., Ang E.L., Ng W.-H., See S.-J., Lim C.T., Chan S., Yeo T.-T. (2007). Improved Median Survival for Glioblastoma Multiforme Following Introduction of Adjuvant Temozolomide Chemotherapy. Ann. Acad. Med. Singap..

[138] Stupp R., Gander M., Leyvraz S., Newlands E. (2001). Current and future developments in the use of temozolomide for the treatment of brain tumours. Lancet Oncol..

[139] Ortiz R., Perazzoli G., Cabeza L., Jiménez-Luna C., Luque R., Prados J., Melguizo C. (2021). Temozolomide: An Updated Overview of Resistance Mechanisms, Nanotechnology Advances and Clinical Applications. Curr. Neuropharmacol..

[140] Lee S.Y. (2016). Temozolomide resistance in glioblastoma multiforme. Genes Dis..

[141] Jiapaer S., Furuta T., Tanaka S., Kitabayashi T., Nakada M. (2018). Potential Strategies Overcoming the Temozolomide Resistance for Glioblastoma. Neurol. Med.-Chir..

[142] Yoshimoto K., Mizoguchi M., Hata N., Murata H., Hatae R., Amano T., Nakamizo A., Sasaki T. (2012). Complex DNA repair pathways as possible therapeutic targets to overcome temozolomide resistance in glioblastoma. Front. Oncol..

[143] Della Monica R., Cuomo M., Buonaiuto M., Costabile D., Franca R.A., Caro M.D.B.D., Catapano G., Chiariotti L., Visconti R. (2022). MGMT and Whole-Genome DNA Methylation Impacts on Diagnosis, Prognosis and Therapy of Glioblastoma Multiforme. Int. J. Mol. Sci..

[144] Sharma S., Salehi F., Scheithauer B.W., Rotondo F., Syro L.V., Kovacs K. (2009). Role of MGMT in tumor development, progression, diagnosis, treatment and prognosis. Anticancer Res..

[145] Cenciarini M., Valentino M., Belia S., Sforna L., Rosa P., Ronchetti S., D’adamo M.C., Pessia M. (2019). Dexamethasone in Glioblastoma Multiforme Therapy: Mechanisms and Controversies. Front. Mol. Neurosci..

[146] Kostaras X., Cusano F., Kline G., Roa W., Easaw J. (2014). Use of Dexamethasone in Patients with High-Grade Glioma: A Clinical Practice Guideline. Curr. Oncol..

[147] Carminucci A., Tejero R., Huang Y., Danish S., Friedel R.H., Foty R. (2020). Teaching an Old Drug New Tricks: Dexamethasone as an In Vivo Inhibitor of Glioblastoma Dispersal. Cureus.

[148] Wang W., Hayashi J., Serrero G. (2006). PC Cell–Derived Growth Factor Confers Resistance to Dexamethasone and Promotes Tumorigenesis in Human Multiple Myeloma. Clin. Cancer Res..

[149] Lo H.-C., Hsu J.-H., Lai L.-C., Tsai M.-H., Chuang E.Y. (2020). MicroRNA-107 enhances radiosensitivity by suppressing granulin in PC-3 prostate cancer cells. Sci. Rep..

[150] Greither T., Steiner T., Bache M., Serrero G., Otto S., Taubert H., Eckert A.W., Kappler M. (2021). GP88/PGRN Serum Levels Are Associated with Prognosis for Oral Squamous Cell Carcinoma Patients. Biology.

[151] Noch E.K., Ramakrishna R., Magge R. (2018). Challenges in the Treatment of Glioblastoma: Multisystem Mechanisms of Therapeutic Resistance. World Neurosurg..

[152] Cohen-Inbar O., Zaaroor M. (2016). Immunological Aspects of Malignant Gliomas. Can. J. Neurol. Sci./J. Can. des Sci. Neurol..

[153] Abella V., Pino J., Scotece M., Conde J., Lago F., Gonzalez-Gay M.A., Mera A., Gómez R., Mobasheri A., Gualillo O. (2017). Progranulin as a biomarker and potential therapeutic agent. Drug Discov. Today.

[154] Bateman A., Cheung S.T., Bennett H.P.J. (2018). A Brief Overview of Progranulin in Health and Disease. Methods Mol. Biol..

[155] Senhaji N., Houssaini A.S., Lamrabet S., Louati S., Bennis S. (2022). Molecular and Circulating Biomarkers in Patients with Glioblastoma. Int. J. Mol. Sci..

[156] Faulkner S., Jobling P., Rowe C.W., Oliveira S.R., Roselli S., Thorne R.F., Oldmeadow C., Attia J., Jiang C.C., Zhang X.D. (2018). Neurotrophin Receptors TrkA, p75NTR, and Sortilin Are Increased and Targetable in Thyroid Cancer. Am. J. Pathol..

[157] Roselli S., Pundavela J., Demont Y., Faulkner S., Keene S., Attia J., Jiang C.C., Zhang X.D., Walker M.M., Hondermarck H. (2015). Sortilin is associated with breast cancer aggressiveness and contributes to tumor cell adhesion and invasion. Oncotarget.

[158] Currie J.-C., Demeule M., Charfi C., Zgheib A., Larocque A., Danalache B.A., Ouanouki A., Béliveau R., Marsolais C., Annabi B. (2022). The Peptide-Drug Conjugate TH1902: A New Sortilin Receptor-Mediated Cancer Therapeutic against Ovarian and Endometrial Cancers. Cancers.

[159] Demeule M., Charfi C., Currie J.-C., Zgheib A., Danalache B.A., Béliveau R., Marsolais C., Annabi B. (2022). The TH1902 Docetaxel Peptide-Drug Conjugate Inhibits Xenografts Growth of Human SORT1-Positive Ovarian and Triple-Negative Breast Cancer Stem-like Cells. Pharmaceutics.

[160] Guo G., Gong K., Puliyappadamba V.T., Panchani N., Pan E., Mukherjee B., Damanwalla Z., Bharia S., Hatanpaa K.J., Gerber D.E. (2019). Efficacy of EGFR plus TNF inhibition in a preclinical model of temozolomide-resistant glioblastoma. Neuro-Oncology.

[161] Luo Z., Wang B., Liu H., Shi L. (2020). TNF Inhibitor Pomalidomide Sensitizes Glioblastoma Cells to EGFR Inhibition. Ann. Clin. Lab. Sci..

[162] Day B.W., Stringer B.W., Al-Ejeh F., Ting M.J., Wilson J., Ensbey K.S., Jamieson P.R., Bruce Z.C., Lim Y.C., Offenhäuser C. (2013). EphA3 Maintains Tumorigenicity and Is a Therapeutic Target in Glioblastoma Multiforme. Cancer Cell.

[163] Wang L., Tang S., Yu Y., Lv Y., Wang A., Yan X., Li N., Sha C., Sun K., Li Y. (2021). Intranasal Delivery of Temozolomide-Conjugated Gold Nanoparticles Functionalized with Anti-EphA3 for Glioblastoma Targeting. Mol. Pharm..

[164] Chen W., Jiang M., Yu W., Xu Z., Liu X., Jia Q., Guan X., Zhang W. (2021). CpG-Based Nanovaccines for Cancer Immunotherapy. Int. J. Nanomed..

[165] Parsons D.W., Jones S., Zhang X., Lin J.C.-H., Leary R.J., Angenendt P., Mankoo P., Carter H., Siu I.-M., Gallia G.L. (2008). An Integrated Genomic Analysis of Human Glioblastoma Multiforme. Science.

[166] Xu J., Xilouri M., Bruban J., Shioi J., Shao Z., Papazoglou I., Vekrellis K., Robakis N.K. (2011). Extracellular progranulin protects cortical neurons from toxic insults by activating survival signaling. Neurobiol. Aging.

